# Precision Neurosurgery: A Path Forward

**DOI:** 10.3390/jpm11101019

**Published:** 2021-10-12

**Authors:** Vianney Gilard, Stéphane Derrey, Stéphane Marret, Soumeya Bekri, Abdellah Tebani

**Affiliations:** 1 Department of Neurosurgery, Normandie University, UNIROUEN, CHU Rouen, INSERM U1245, 76000 Rouen, France; stephane.derrey@chu-rouen.fr; 2 Department of Neonatology, Pediatric Intensive Care, and Neuropediatrics, Normandie University, UNIROUEN, CHU Rouen, INSERM U1245, 76000 Rouen, France; stephane.marret@chu-rouen.fr; 3 Department of Metabolic Biochemistry, Normandie University, UNIROUEN, INSERM U1245, CHU Rouen, 76000 Rouen, France; abdellah.tebani@chu-rouen.fr (A.T.); soumeya.bekri@chu-rouen.fr (S.B.)

**Keywords:** neuro-oncology, neurosurgery, precision medicine, personalized medicine, omics, medical imaging

## Abstract

Since the inception of their profession, neurosurgeons have defined themselves as physicians with a surgical practice. Throughout time, neurosurgery has always taken advantage of technological advances to provide better and safer care for patients. In the ongoing precision medicine surge that drives patient-centric healthcare, neurosurgery strives to effectively embrace the era of data-driven medicine. Neuro-oncology best illustrates this convergence between surgery and precision medicine with the advent of molecular profiling, imaging and data analytics. This convenient convergence paves the way for new preventive, diagnostic, prognostic and targeted therapeutic perspectives. The prominent advances in healthcare and big data forcefully challenge the medical community to deeply rethink current and future medical practice. This work provides a historical perspective on neurosurgery. It also discusses the impact of the conceptual shift of precision medicine on neurosurgery through the lens of neuro-oncology.

## 1. Introduction

Neurosurgery is considered the oldest surgical practice. It has been reported that the first trepanations were performed in the Neolithic period and traces of human trepanations dating back to 7000 BC have been found with bone consolidation around the drill hole, proof of the survival of these first patients [1,2]. Despite its long history, neurosurgery has been able to rely on the great technological advances of its time. In recent years, with the emergence of omics-based biological lenses, precision medicine has emerged as one of the great advances in various fields of medicine [3,4]. Its contribution can be found in the field of diagnosis with the development of biomarkers, but it also provides prognostic elements and opens the way to new targeted therapies [5]. Neurosurgery is no exception and precision, or personalized, medicine is present in our daily surgical practice. Precision medicine (PM) aims to place the patient at the centre of the healthcare pathway by integrating individual medical and biological data while taking into account the great diversity between individuals. These new approaches invite us to reinvent ourselves as neurosurgeons and to question our practice and profession. In line with the spirit and training of our mentors, engineering, imaging and molecular biology techniques have become increasingly familiar components of the operating room. They could not replace the fundamental principles of surgery, which are the core of the operative indication, including a rigorous knowledge of anatomy and flawless dexterity painstakingly earned through relentless and long cooperative practice. Nevertheless, these techniques have become very useful in providing high quality and safe care to our patients. One of the fields that best illustrates the contribution of new technologies and interdisciplinarity to neurosurgery is undoubtedly neuro-oncology. In the following article, we aim to provide historical perspective and discuss how neurosurgery is evolving towards its next deep technological shift by fully embracing precision medicine, particularly in the field of neuro-oncology.

## 2. Neurosurgery: A Short Historical Perspective

Even though the history of neurosurgery seems to begin in the Neolithic period, 5000 years later, it was the pre-Inca civilizations that developed trepanning techniques. The indications of these trepanations were very different from today and had a religious or magical tone [2,6]. Nevertheless, these procedures were also performed in the context of cranial trauma, headaches, epilepsy and behavioural disorders. Later, the Incas raised their practice of cranial surgery to the rank of art. This is particularly highlighted by the diversity of their metal-based instruments [7,8]. By looking at the survival rate following cranial surgery throughout history, Kushner et al. [2] showed that the Incas had a survival rate of 75% compared to 45% during the American Civil War. The main cause of mortality at the time was infection, but the Incas applied ointments and metal prosthesis, ancestor of the current cranial vault plasties [7,8]. However, solely technical skills are not enough to provide quality surgery. Understanding brain physiology is, obviously, important for treating it more effectively. The first Greek physicians helped to better understand these concepts. Hippocrates in the fourth century BC [9,10], who is considered the father of medicine, explained in his *Omnia Opera Hippocratis* that the knowledge of physiology allowed for a better understanding of manual technique and that the technique alone was not enough to guarantee high quality surgery. Galen, in the second century A.D., elaborated on Hippocrates’ work, in addition to perfecting anatomical knowledge through the observation of a large number of subjects and taking care of injured gladiators [11]. Although ancient Greece practices, such as trepanations, were refined over the centuries, endocranial surgery as such did not appear until the 16th century. The cruelty of war allowed significant medical leaps in the Middle Ages and the Renaissance. One of the leading figures of modern surgery was undoubtedly Ambroise Paré (1510–1590) [12]. He perfected the trepanation techniques and vessel ligation and introduced the notion of asepsis by using red iron cauterisation. A historical convergence between people’s lives and neurosurgery is the death of Henry II of Navarre, who died of an infection following a craniocerebral wound during a tournament. Ambroise Paré, who performed the autopsy, described a cerebral empyema as the cause of death. Across the Channel, the pioneer of modern neurosurgery was Sir Victor Horsley (1857–1924) [13,14]. He was a military surgeon working at the Queen Square Hospital in London. With the support of his neurophysiologist colleagues, he hypothesized that epilepsy was a disease of the cerebral cortex and that its resection could stop the development of epilepsy. Based on clinical observations, he performed the first cerebral cortectomies on three patients. This procedure, although supported by imaging and physiological investigations, is commonly performed today [6,7]. Harvey Cushing (1869–1939) [15] is the first American neurosurgeon to operate on brain tumours. He performed more than 2000 brain surgeries and the description of his approaches led to the improved survival of neurosurgical patients. Unfortunately, surgery still carries the burden of its own history. From the 12th to the 18th century, barber-surgeons were distinct from physicians. The medical progress made in parallel with surgical practice only slightly enriched surgical know-how. The main pitfall of surgery is asepsis, as is also the case for neurosurgery. The birth of modern post-war neurosurgery was made possible through progress in perioperative conditions (asepsis and anaesthesia) and led to a better understanding of pathology and progress in imaging and neurophysiology.

## 3. Advances in Neurosurgery: First Steps towards Precision Surgery

Very early on, neurosurgeons defined themselves as physicians with a surgical practice. “Neurosurgery is not only the art of removing tumours from the brain, but it is the means to learn in a precise way the functions of the human brain” once said the French neurosurgeon Clovis Vincent (1879–1947) [16]. He was the first physician in charge of a dedicated surgical department. In Canada, Wilder Penfield (1891–1976) [17], also a neurologist and a neurosurgeon, described cerebral somatotopy. He contributed, as did Clovis Vincent, a student of Babinski (1857–1932), to the description of memory circuits. Based on his work, Harvey Cushing performed the first intraoperative stimulations to explore the functions of the resected brain areas. These historic explorations prefigured the development of awake surgery. One of the aspects of modern neurosurgery most related to these technical advances is certainly functional neurosurgery. It aims at adjusting certain functions of the neuraxis when drug treatment is insufficient or not recommended. Classically, it includes deep brain stimulation, epilepsy surgery, pain surgery and radiosurgery. The pioneers come from various backgrounds. Lars Leksell (1907–1986) was a Swedish physicist and neurosurgeon [18]. He is credited with one of the first stereotaxis frameworks and the invention of radiosurgery. In France, Jean Talaraich (1911–2007) was a psychiatrist and then a neurosurgeon [19]. With Jean Bancaud (1921–1993), a neurologist, they wrote the first stereotaxis atlas, which remains a reference work. They proposed a stereotaxis framework that allowed for the implantation of electrodes and brain biopsy with flawless accuracy. Surgical precision and accuracy are at the core of precision medicine because it relies on the development of engineering techniques. These surgical techniques were developed before the brain scanner (in the 1970s). The anatomical landmarks were based on statistical charts derived from hundreds of cadaveric observations and indirect landmarks. These charts represent precious information—stored, quantifiable, standardized and ready-to-use to assist the surgical act. Conceptually, these charts are the precursors to current informational databases of anatomical, clinical and biological big data. In 1972, Godfrey Hounsfield (1919–2004) [20] was awarded the Nobel Prize in Medicine for the discovery of the first computerized tomography (CT) scan. It was a technological breakthrough for neurosurgery. For the first time we could, safely and non-invasively, see the brain directly without opening the skull. The scanner resolution progressively improved and the surge of magnetic resonance imaging (MRI) in the early 1990’s allowed for a cleaner, non-irradiating image. In only three decades, MRI has gone from innovation to a common practice. At the same time, progress in optical magnification and neuro-endoscopy techniques led to advances in operative approaches, dissection management or hemostasis, and even new interventions (i.e., endoscopic ventriculocysternostomy) [21]. At the dawn of this millennium, robotics entered the operating room. Surgical robots (for cerebral or spinal stereotaxis for the moment), navigation and even augmented reality systems are used on a daily basis. More recently, this integration of human and technical progress has been enriched by advances in molecular biology and substantially contributes to the development of multimodal techniques paving the way to precision surgery [22].

## 4. Precision Medicine in Daily Neuro-Oncology

Neurosurgery has always cultivated interdisciplinarity as described above. Some of its prominent members came from different backgrounds. Lars Leksell for example was a physicist by training. So too was Jean Talairach. The different branches of neurosurgery have been enriched by their respective backgrounds. With the advent of molecular biology techniques, precision medicine is no longer an outsider to the operating room but has become an active component. The most striking example is certainly neurosurgical oncology. This is particularly highlighted through glioblastoma. Indeed, its management has made significant advances [23,24]. Glioblastoma, although the most common primary malignant tumour of the central nervous system, still lacks curative strategies. Its prognosis remains poor, even in case of complete surgical resection with a median survival of 15 months [25]. One of the reasons for treatment resistance in glioblastoma is its heterogeneity. Thus, the integration of molecular biology, genomics and more recently metabolomics and transcriptomics data are of interest and has led to a better understanding of glioblastoma heterogeneity and biological plasticity [26,27]. The digital revolution and the multimodal big data surge have made it possible to characterize this tumour in order to better understand its genesis, clinical heterogeneity, functional effects and the reasons underlying its resistance to treatment [28,29]. The main goals of precision medicine are to better understand glioblastoma signalling biological networks and to define common and distinct pathways that could explain its clinical heterogeneity. This will allow for biomarker and prognostic discovery to open the way to targeted and individualized therapies by targeting an alteration in these signalling pathways [30,31].

Historically, the diagnosis of glioblastoma was based on its WHO classification, which originally derived from histological criteria alone. The latest WHO classification of 2016 has integrated the latest molecular biology data as well as prognostic elements and responses to treatment related to its metabolic characteristics [32]. Among the new features of the 2016 WHO classification is the mutation status (mutated or wild type) of the *IDH1* and *IDH2* genes. It is postulated that *IDH*-mutated gliomas inhibit tumour suppressor oncogenes, inducing the development of gliomas. Consequently, *IDH*-mutated status is an important prognostic factor for glioblastomas [33]. The second component of classification of glial lesions is the presence or absence of a 1p/19q codeletion. The 1p/19q co-deletion corresponds to the complete loss of 1p and 19q secondary to a translocation. It is associated with a better prognosis and a better response to chemotherapy for grade II and III gliomas [34]. Another recently described signalling pathway in glioblastoma is the telomerase-related senescence escape pathways through mutation of the *ATRX* (alpha thalassemia/mental retardation syndrome X-linked) gene or *TERT* (telomerase reverse transcriptase) promoter [27]. The *ATRX* gene was initially discovered in patients with X-linked mental retardation. The ATRX protein is a chromatin remodelling protein whose main role is to maintain genomic stability. Thus, in case of *ATRX* variation, there is a telomerase lengthening allowing glial cells to escape senescence. *ATRX* variation is a prognosis marker, rarely observed in *IDH*-wild type glioblastoma. Variants in the *TERT* promoter or *ATRX* gene are present in 90% of mutated *IDH* gliomas but are rarely associated. In recent series [35,36], it appears that the *TERT* promoter gene mutation is associated with poor outcomes in *IDH* wild type glioblastoma and favourable outcomes in *IDH* mutated glioblastoma. The prognostic value of the *TERT* promoter status is discussed due to its high frequency and confounding factors. For example, the association with other molecular alterations such as O(6)-methylguanine-DNA methyltransferase (MGMT) methylation is of interest. MGMT is an enzyme that repairs O6-methylguanine of DNA to guanine. It protects the tumour cell against cytotoxic damage from alkylating chemotherapies, such as temozolomide [37,38]. As a consequence, methylation of the *MGMT* promoter leads to a decrease in MGMT expression and in the repair of temozolomide-induced lesions. This status is a predictive marker for a better response to alkylating agents and an independent favourable prognostic marker in glioblastoma. Variants in genes encoding histones, which were first identified in childhood glioma, are a promising avenue for a better understanding of glioma signalling pathways [35]. Indeed, these variants are frequently found in various cancers and could contribute to their epigenetic regulation. The heterogeneity of tumours and the large number of molecular biology discoveries yielded through omics highlights the need for more personalized knowledge through integrative strategies to achieve precision medicine.

The search for a single biomarker for glioblastoma has been the focus of sustained research for many years [39]. Medical imaging and MRI spectroscopy, in particular, were mainstream strategies in clinical practice [40]. It mainly consists in highlighting specific metabolic patterns in the tumour tissue. The development of this technique required a large number of patients. The patient is, in this case, his own control, because the healthy brain parenchyma, usually contralateral, is used as a control tissue. Thus, MR spectroscopy in glioblastomas is characterized by a specific increase in choline/N-acetylaspartate and choline/creatinine ratios [41]. In addition, an elevated peak of lactate and lipids as well as a decreased peak of myoinositol are reliable information for the diagnosis of glioblastoma. With the advent of big data, radiological diagnosis continues to progress. Recently, researchers at the University of Washington proposed an algorithm based on artificial intelligence and deep learning [42]. Their software has proven effective in diagnosing six categories of brain tumours from conventional MRI sequences. This radiological marker cannot, currently, replace a histological analysis. This highlights the urgent need for a sensitive blood biological signature. The use of large biobanks has allowed for multi omics studies to propose biomarkers, defined as a clinical, radiological and biological signatures, that allow the definition of sub-groups of glioblastomas [43,44]. This refers to liquid biopsies whose aim is to propose a diagnosis through a simple blood sample. Liquid biopsy has the advantage of avoiding an invasive procedure, such as brain biopsy, and easily repeatability [29,45,46]. However, the development of such techniques has pitfalls even though the concept is elegant. First, the heterogeneity of glial tumours makes it difficult to find a single biomarker. Secondly, the blood–brain barrier is a powerful obstacle to the discovery of a blood biomarker of an intraparenchymal processes. Finally, an ideal biomarker must allow for monitoring of therapeutic efficacy or relapse. However, therapeutics can modify this biomarker. Thus, despite sustained research, there is currently no clear blood biomarker for the diagnosis and follow-up of glioblastoma patients. Nevertheless, recent analyses have highlighted genes or metabolites of interest in the diagnosis of glioblastoma [45,47,48]. Some of them are also prognostic indicators and might pave the way to potential targeted therapies.

## 5. Towards a Precision Surgery

Despite the above-mentioned progress, it remains in question whether we will see the birth of precision surgery. At the dawn the current century, the main progress in neurosurgery has been based on intraoperative navigation to better locate the tumour from preoperative imaging [49]. Awake surgery has increased the resection rate by decreasing the morbidity of the surgical procedure [50]. At present, imaging is performed during surgery and allows for resection assessment in real time [51]. Augmented reality techniques allow for the smooth integration of these imaging data into the surgeon’s microscope to directly visualize the lesion and the peri-tumour area. More recently, intraoperative analysis of resected tissue has been made possible by the miniaturization of omics techniques. Thus, some teams have tested the integration of a mass spectrometer in the cavitronic ultrasound aspirator [52,53]. This allows us to limit the resection to the tissue invaded by the tumour. One of the difficulties of this technology is the definition of the metabolites of interest. These are different depending on whether they concern the fleshy portion of the tumour, a possible cystic portion, the contrast medium or the peri-tumoral zone. Secondly, the contribution of mass spectrometry cannot be free of the functional limits linked to the tumour resection. The surgeon’s hand is often stopped by the risk of morbidity of a resection. Nevertheless, in situ metabolic phenotyping is a definite contribution and is a complement to navigation and microscopy techniques. The miniaturization of these advanced imaging technologies makes their clinical use possible and opens the door to a new way of understanding the procedure guided by molecular biology data. Conceptually, surgical precision and technological development are only two faces of the same coin: precision surgery. One of its fundamental principles is to put the patient at the centre of healthcare in order to take into account individual and specific biological and clinical attributes [54,55,56]. To do so, it is necessary to differentiate the normal from the pathological, which relies on the development and analysis of large biobanks integrating various multimodal data. It is worth noting, however, that in the current technological and regulatory state-of-the-art, these technological shifts cannot yet replace surgical decision, which is still solely guided by the surgeon’s experience and dexterity.

## 6. Conclusions

Neurosurgery is considered one of the oldest surgical disciplines. Since ancient times, it has been able to embrace major technological advances for the benefit of patients. Its progress has been made possible by the knowledge of anatomy, the development of radiology, microscopy, robotics and, recently, big data. Indeed, the development of data-driven medicine has considerably modified clinical practice, in neuro-oncology in particular. This has allowed a better understanding of pathogenesis and thus opened the way for innovative personalized therapies and disease management. The future of neurosurgery, and medical practice in general, lies in the smooth integration of multidisciplinary perspectives in both translational and clinical neurosurgery to achieve the promise of precision surgery.

## Data Availability

Not applicable.
